# Use of MALDI-TOF mass spectrometry and IDBac to mine for understudied bacterial genera from the environment

**DOI:** 10.1093/ismeco/ycaf046

**Published:** 2025-03-13

**Authors:** Antonio Hernandez, Nyssa K Krull, Brian T Murphy

**Affiliations:** Department of Pharmaceutical Sciences, College of Pharmacy, University of Illinois at Chicago, Chicago, IL 60607, United States; Center for Biomolecular Sciences, College of Pharmacy, University of Illinois at Chicago, Chicago, IL 60607, United States; Department of Pharmaceutical Sciences, College of Pharmacy, University of Illinois at Chicago, Chicago, IL 60607, United States; Center for Biomolecular Sciences, College of Pharmacy, University of Illinois at Chicago, Chicago, IL 60607, United States; Department of Pharmaceutical Sciences, College of Pharmacy, University of Illinois at Chicago, Chicago, IL 60607, United States; Center for Biomolecular Sciences, College of Pharmacy, University of Illinois at Chicago, Chicago, IL 60607, United States; Institute for Tuberculosis Research, College of Pharmacy, University of Illinois at Chicago, Chicago, IL 60612, United States

**Keywords:** MALDI, mass spectrometry, IDBac, bacteria, natural products, database

## Abstract

Bacterial natural products have greatly contributed to the global drug discovery effort. Further, the incorporation of understudied bacterial taxa into discovery pipelines remains a promising approach to supply much needed chemical diversity to this effort. Unfortunately, researchers lack rapid and efficient techniques to accomplish this. Here we present an approach that employs matrix-assisted laser desorption/ionization time of flight mass spectrometry (MALDI-TOF MS) and the bioinformatics platform IDBac to perform targeted isolation of understudied bacteria from environmental samples. A dendrogram of MS protein spectra from 479 unknown bacterial isolates was seeded with spectra from 50 characterized strains that represented target understudied genera. This method was highly effective at identifying representatives from target taxa, demonstrating an 86.3% success rate when an estimated genus level cutoff was implemented in the dendrogram. Overall, this study shows the potential of MALDI-MS/IDBac to mine environmental bacterial isolate collections for target taxa in high-throughput, particularly in the absence of proprietary software. It also provides a cost-effective alternative to morphology and gene-sequencing analyses that are typically used to guide identification and prioritization strategies from large bacterial isolate collections.

## Graphical Abstract

**Graphical Abstract:**
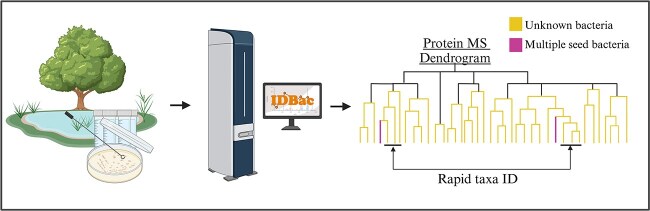

## Introduction

Natural products and their semisynthetic derivatives comprise a major portion of the available therapeutic arsenal [1]. In particular, bacterial natural products represent nearly 90% of available clinical antibiotics [2–4]. However, high rediscovery rates of known chemical scaffolds have hampered this field in the past few decades. This trend has occurred simultaneously with rising rates of resistance to therapeutics and has necessitated access to areas of new chemical space to supply the next generation of drug leads [5]. Whereas new chemical space can be produced by understudied phylogenetic space [6–13], researchers lack rapid and efficient techniques to identify understudied bacteria in the front end of discovery pipelines [14].

Genetic evidence supports that novel natural products are encoded within understudied phylogenetic space, arbitrarily defined here as taxa that contain genome sizes of ~5 Mbp or larger and have fewer than 20 published natural products. For example, phyla such as Planctomycetota [15–18], Bacteroidota [19, 20], and Acidobacteriota [7, 21], among others, encode for the production of a large diversity of unique secondary metabolites that share low protein homology to biosynthetic gene clusters of more heavily investigated phyla. Though even within the phyla Actinomycetota and Pseudomonadota, which harbor some of the most well studied genera to date (i.e. *Streptomyces* and *Pseudomonas* spp., respectively), there remain genera that have high biosynthetic potential with few or no reported natural products [8, 19, 22, 23].

Together this body of evidence suggests that mining the environment for understudied bacterial taxa represents a promising strategy to discover novel natural product space. Here we present a blueprint that utilizes matrix-assisted laser desorption/ionization time of flight mass spectrometry (MALDI-TOF MS) and the bioinformatics platform IDBac [24–26] to target the isolation of understudied bacteria from environmental samples.

### Use of MALDI-TOF MS to identify bacterial isolates.

Since the late 1990’s, researchers have established MALDI-TOF MS as a technique to identify bacteria through matching mass spectra fingerprints of unknown isolates to spectra of genetically verified bacterial strains. Broadly speaking, spectral similarities between phylogenetically related bacteria are largely driven by ionized ribosomal proteins in the range of 2,000 to 20,000 Da [27–29]. Utilizing this principle, two commercial databases and accompanying software were approved by the FDA to expedite bacterial identification in clinical settings (MALDI Biotyper, 2013 and Vitek MS, 2017). While this has led to improved diagnostics and may offer a long-term cost savings approach for large hospital systems, these platforms are cost-prohibitive for many researchers outside of the medical field [30, 31]. Some academic labs have utilized this approach to identify bacterial isolates and document microbial composition and diversity [32], however the majority of these studies have focused on clinical pathogens or represent limited taxonomic diversity [27–29, 33–37]. Related to the current work, a few studies have created custom databases of protein MS spectra, associated them with the MALDI Biotyper database, and used this to identify specific bacteria from the environment [38–42].

Prior to 2018, the non-clinical research community lacked open access infrastructure to process and visualize similarities within both protein (2000–20 000 Da) and natural product (200–2 000 Da) mass ranges from large numbers of isolates. Generally speaking, available software at that time allowed researchers to search individual spectra against a database to obtain a putative species identification on individual isolates. However, either these packages were not designed to accommodate hundreds to thousands of bacterial isolates at a time, or the software was proprietary. These roadblocks prevented analyses that sought to visualize complex relationships within a strain or isolate collection. To address this, our team developed the open-source bioinformatics tool IDBac [24–26]. IDBac allows users to first cluster bacterial isolates by protein spectrum similarity, followed by subgrouping them based on feature overlap within corresponding natural product spectra, a function not present in existing platforms [26]. The capacity to process and compare large datasets was an essential prerequisite toward addressing what is currently a major challenge in the field of bacterial natural product drug discovery—the ability to rapidly mine for multiple understudied taxa and dereplicate known taxa from a collection of environmental bacteria.

Our team previously detailed the use of a “cut” across a dendrogram to form discrete groups within a diverse collection of environmental isolates and noted limitations of employing hierarchical clustering to group bacterial isolates above the taxonomic level of genus [43]. In the absence of a comprehensive protein MS database that can be used to identify an isolate to species or subspecies level, it was necessary to define a cut height within the dendrogram that would approximate genus-level clustering of the diverse array of bacterial taxa present. Accomplishing this goal would allow for the ‘seeding’ of spectra of characterized bacterial isolates into a dendrogram in order to rapidly identify multiple bacterial genera at a time.

**Figure 1:**
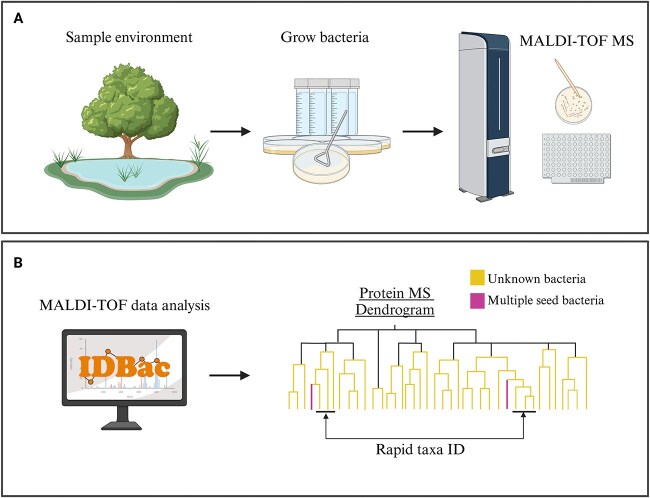
**MALDI-TOF MS workflow.** (A) Bacteria are grown from environmental samples. Cell mass from individual colonies is then plated onto a MALDI target plate data are collected in the spectrometer. (B) MALDI data are uploaded into IDBac, which generates a dendrogram of MS protein spectra from the bacterial isolates. Created in BioRender: https://BioRender.com/x11a535.

In the method described herein (Fig. 1), MALDI-TOF MS composite spectra of characterized target strains are ‘seeded’ into a dendrogram of tens to thousands of composite spectra from unknown environmental isolates. Investigation of seed groupings allowed the rapid identification of target genera with high accuracy. Importantly, only MS spectra from target strains are needed to carry out this method; a diverse database of bacterial MS spectra is not required. This approach puts the identification of talented taxa early in the natural product discovery process. It takes advantage of the throughput, experimental simplicity, and data visualization capacity of MALDI MS/IDBac to overcome a major bottleneck in the discovery pipeline—the relative impracticality, high cost, and time commitment of performing 16S rRNA sequencing on hundreds to thousands of environmental isolates.

## Materials and methods

### Sample collection and processing.

Environmental samples were collected through collaboration as well as intra-lab collection expeditions from 2021 to 2022. Thirteen collection efforts spanning the United States (Table S6) yielded 44 samples, including: soil, moss, lichen, cave lake water, freshwater and marine macroalgae, freshwater and marine sponge, and forest detritus. Prior to plating, solid samples were vortexed with 25 ml of sterile deionized water three times to remove external debris. Additionally, sponge samples were homogenized as described by Clark et al [43]. Following sample preparation, 100 μl of four serial dilutions were plated on five solid-substrate media plates, each containing varying nutrient levels and cycloheximide (28 μM) to inhibit fungal growth (Table S7). Inoculated diversity plates were sealed with Parafilm® and incubated at ambient temperature for 1–10 weeks. From these plates, all discernable colonies were purified on their corresponding media and axenic cultures were used for downstream MADI-TOF MS analysis and DNA extraction. A total of 479 isolates from this collection were used in this study.

### MALDI-TOF MS analysis.

Following purification, each isolate was transferred to A1 agar media and incubated for 7 days in preparation for MALDI-TOF MS data acquisition. Using a sterile toothpick, a small portion of isolated colonies was smeared within designated spots of a 384-spot MALDI target plate (Bruker Daltonics, Billerica, MA, USA), forming a thin cell-mass layer. After bacterial isolates were smeared, 1 μl of 70% formic acid (7:3 Optima, Fisher Chemical: Optima LC-MS Grade Water Fisher Chemical) was added to each well. Matrix was prepared using α-cyano-4-hydroxycinnamic acid (powder, 98% pure, Sigma-Aldrich, part-C2020) 50% acetonitrile, 47.5% water, and 2.5% trifluoroacetic acid. All solvents used were LC-MS grade. Once the formic acid evaporated, 1 μl of 10 mg/ml α-cyano-4-hydroxycinnamic acid matrix was pipetted onto each bacterial smear. Spectra from three to eight biological replicates were collected per isolate (dependent on the collection expedition). A detailed description of this procedure was previously published [24].

Following data acquisition, mass spectra were manually checked for quality by examining the intensity, signal to noise, and peak shapes of each mass spectrum. Sample spectra can be found in Fig. S3. Once quality controls were passed, IDBac was used to generate consensus spectra that were used for the analysis. Regarding seed strains used in this study, genera were selected from our strain collection based on the criteria previously described: ~5 Mbp genome sizes or larger and fewer than 20 published natural products. This dataset is not intended to be a comprehensive list of understudied bacterial genera. Each seed was grown on high nutrient agar at room temperature for roughly seven days.

MALDI-TOF MS analyses were carried out using two instruments. One instrument was an Autoflex Speed LRF mass spectrometer (Bruker Daltonics) equipped with a smartbeam™-II laser (355 nm). Automated data acquisitions were performed using flexControl software v. 3.4.135.0 (Bruker Daltonics) and flexAnalysis software v. 3.4. Protein spectra were recorded in positive linear mode (1200 shots; RepRate: 2000 Hz; delay: 29731 ns; ion source 1 voltage: 19.5 kV; ion source 2 voltage: 18.35 kV; lens voltage: 7 kV; mass range: 1.92 kDa to 21 kDa, matrix suppression cutoff: 1.9 kDa). The second instrument employed was a rapifleX MALDI Tissuetyper mass spectrometer (Bruker Daltonics) equipped with a smartbeam™ 3D laser (355 nm). Automated data acquisitions were performed using flexControl software v. 4.0.46.0 (Bruker Daltonics) and flexAnalysis software v. 3.4. Protein spectra were recorded in positive linear mode (2000 shots; RepRate: 5000 Hz; delay: 28272 ns; ion source 1 voltage: 20 kV; ion source 2 voltage: 18.45 kV; lens voltage: 9 kV; mass range: 2 kDa to 19.99 kDa; matrix suppression cutoff: 1.6 kDa). Protein spectra were corrected with external Bruker Daltonics bacterial test standard (BTS).

### Sample analysis using IDBac

IDBac version 1.1.10 was used for analysis (https://chasemc.github.io/IDBac/) [26]. The analysis settings to generate the dendrogram are as follows: Percent presence = 70; Signal to Noise Ratio = 4; Lower Mass Cutoff = 3000 Da; Upper Mass Cutoff = 15000 Da; ppm tolerance = 500. The clustering settings used in this analysis are as follows: Distance Algorithm = cosine; Clustering Algorithm = Ward; Presence/Absence was used as well. IDBac uses various R packages to operate [26]. The most current version of IDBac can be found at: https://idbac.org/.

### DNA extraction and 16S rRNA gene amplification and analysis

DNA was extracted from 74 isolates using a modified colony Polymerase chain reaction (PCR) protocol [44]. Axenic cultures were incubated in 100 μl of 1x IDTE pH 8.0 for 5 minutes at 90°C. The mixture was then centrifuged at 10,000 RPM for ~2 minutes and 3 μl of the supernatant was transferred to a 0.2 ml Eppendorf tube containing: 8.5 μl molecular grade water, 12.5 μl KAPA2G Robust HotStart ReadyMix, and 0.5 μl of 16S rRNA universal primers 27F (5′-AGA GTT TGA TCC TGG CTC AG--3′) and 1492R (5′- GGT TAC CTT GTT ACG ACT T-3′) [45]. PCR conditions included a primary denaturation at 95°C for 5 minutes then 35 cycles of denaturation at 95°C for 30 seconds, annealing at 60°C for 30 seconds, extension at 72°C for 1 minute with a final elongation at 72°C for 5 minutes. PCR product purification and Sanger sequencing were performed by Eurofins Genomics (eurofinsgenomics.com/en/products/dna-sequencing). Pairwise alignment, generation of consensus sequences and Basic Local Alignment Search Tool (BLAST) analysis were conducted using Geneious Prime software Version 2023.2.1. Taxonomies were assigned based on >98% identity and pairwise alignment with strains in the NCBI Genbank database.

## Results and discussion

### Selection of bacterial seed spectra

To test the ability of MALDI-TOF MS/IDBac to simultaneously mine for multiple understudied bacterial genera, a seed dataset of 50 understudied environmental strains was selected from our in-house collection (see Materials and Methods for more details). These strains were previously identified as understudied via either 16S rRNA gene sequencing or whole genome sequencing and span 5 phyla and 24 genera (Tables S1, S2). In addition to these seeds, we incorporated spectra from 18 previously characterized strains (via 16S rRNA analysis) that spanned four genera (Table S1). Although the 18 strains were not part of the understudied genera to be targeted, these were added to provide further definition to the dendrogram; see “Determination of dendrogram cut height” section for details. Spectra of the 50 understudied seeds and 18 well-characterized strains were compiled into a single SQLite file using IDBac and incorporated into a protein dendrogram of unknown environmental bacteria from several collection expeditions.

### Use of MS seed spectra to mine collections of unknown environmental isolates

Collections of bacterial isolates were sourced from thirteen sample collection expeditions that took place between 2021 and 2022. Environmental samples were collected from a mixture of aquatic and terrestrial environments throughout the United States and include (but are not limited to): marine tunicates, freshwater and marine sponges and sediment, soil, moss, and macroalgae. Sample preparation and cultivation varied across collections, though subsequent isolation procedures were followed as described by Clark *et al.* [46]. Briefly, distinguishable colonies were transferred to a high nutrient medium and a small mass of axenic culture was used for MALDI-TOF MS data acquisition [24, 26]. From these data, a protein dendrogram was generated. The dendrogram was created using Ward clustering and cosine distance measurements [47, 48]. Seed spectra from 50 understudied bacteria were integrated into each dendrogram as a single SQLite file. Using Figtree v1.4.4, the dendrogram was normalized to a height of 100 and potential matches were manually assessed.

### Determination of dendrogram cut-height to estimate genus-level identity within groupings

**Figure 2:**
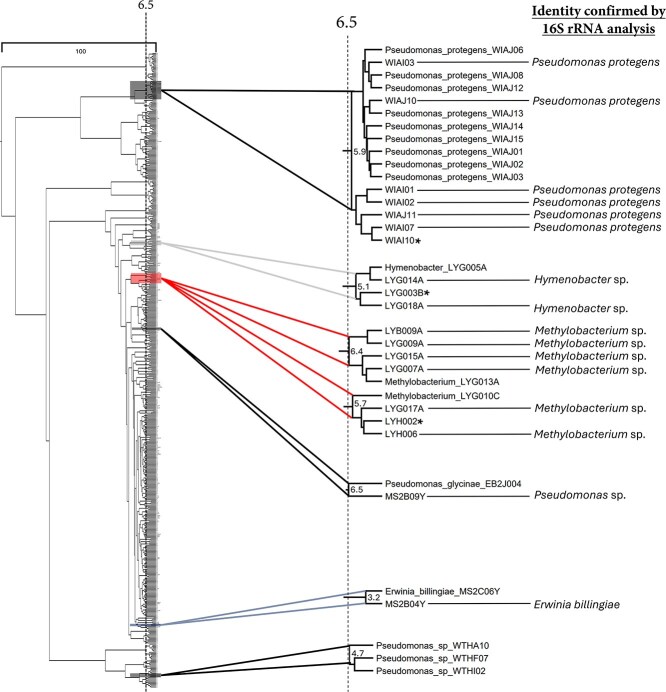
**Use of characterized seed spectra to establish approximate genus level cutoff.** Deciphering phylogenetic relatedness based on MALDI-TOF MS dendrogram groupings is a current challenge within the field. To address this, a dendrogram of 479 unknown isolates from 13 collection expeditions was seeded with spectra from 50 strains representing understudied genera and 18 strains from characterized, more well-studied genera. Observing where and how the seeds grouped allowed for the estimation of a genus-level cutoff. Given the observed groupings, it was predicted that any isolates that grouped at or within the 6.5 cut-off would be associated with the same genus (selected examples appear in Fig. 2). Any isolates that grouped above a parent node of 6.5 were not considered to contain pertinent information. *16S rRNA gene sequencing analysis was inconclusive due to cryostock contamination, therefore a reliable isolate ID was not possible.

Strejcek et al developed a cosine similarity threshold to delineate bacterial species in the absence of a protein MS database [49]. The current approach uses open access software to perform a similar task and can analyze hundreds of isolates simultaneously through the ‘seeding’ of spectra into a dendrogram. We first normalized the dendrogram using a scale of 0-100. Preliminary manual analysis of seed groupings suggested a rough approximation for genus level discrimination at a height of 6.5 (Fig. 2, Table S3). Therefore, it was predicted that any isolates that grouped at or within the 6.5 cut-off would be associated with the same genus. Additional cuts were made at heights 15 and 4; a height of 6.5 provided the most effective genus-level identification of isolates.

The adjusted Wallace coefficient was then employed to assess the accuracy of the aforementioned cut heights to predict genus level clustering [50]. The computed index supported our findings showing a 93% chance that strains within a cluster at a 6.5 cut height are members of the same genus, while a cut height of 15 dramatically decreased the probability to 5%. Notably, a cut height of four yielded the highest fidelity of genus clustering (97%), though this improvement is marginal and fails to capture adjacent members of the same genus that are observed at a 6.5 height (Table S4).

For example, three groups of *Pseudomonas* seed strains were observed in three different sections throughout the dendrogram at heights of 6.5, 5.9, and 4.7 (Fig. 2, Fig. S1). Other seed genera were manually inspected and behaved similarly, indicating that it was acceptable to move forward using the 6.5 cutoff. Importantly, it is common to observe a single genus forming multiple groupings within a dendrogram, as MALDI MS similarity analysis contains a higher degree of resolution than a phylogenetic tree based on 16S rRNA gene sequences [51]. MALDI MS similarity groupings are predominantly based on highly abundant cytosolic ribosomal proteins, however it is likely that other housekeeping proteins (heat shock proteins, DNA binding proteins, and RNA chaperone proteins) contribute to higher resolution clustering of bacterial genera [28, 52].

**Figure 3:**
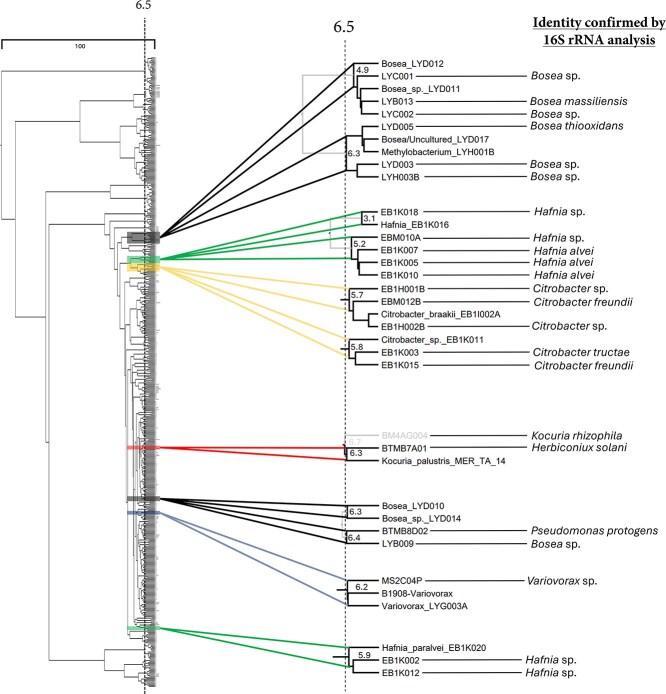
**Dendrogram highlighting environmental isolates that matched with seeds from understudied genera.** This dendrogram highlights instances where seeds from understudied bacterial genera matched with one or more environmental isolates at or within the 6.5 cutoff. Isolates that resided in the same grouping as a seed were identified via 16S rRNA gene sequencing analysis. 86.3% of these isolates accurately matched with the genus of the seed in their respective groupings.

In total, 38 seeds grouped with environmental isolates at or below the 6.5 cutoff (20 seeds from understudied genera and 18 seeds from other previously characterized genera). These represented 18 individual dendrogram groupings (Table S3). Although several taxa correctly grouped using the 6.5 cutoff, there were exceptions. For example, a grouping at 6.3 contained one seed “*Kocuria_palustris*_MER_TA_14” that paired with an environmental isolate from the same order, identified as *Herbiconiux solani* (Fig. 3). However, a slightly higher parent node of 6.7 indicated an additional pairing to an environmental isolate identified as *Kocuria rhizophila*. Three other examples also exhibited the grouping of a seed to an environmental isolate from a different genus at or below the 6.5 cut-off (Fig. S2). While the discrepancies between parent node heights are notable, they are not necessarily an indication of incorrect groupings, rather they highlight the added complexity of MALDI groupings and the need for further investigation since spectra represent ionized proteins from the whole cell mass that have not traditionally contributed to the taxonomic identification of a strain.

With these examples in mind, it is important to note that 6.5 is not a universal genus-level cut height. Groupings near either side of the cutoff are worthy of closer evaluation, depending on the level of accuracy required for a particular study. The cutoff is highly dataset dependent and will vary with each collection of isolates. Among other factors, one major influence on this is the phylogenetic diversity—both genus level diversity and higher—within a dataset, and seed library used. These factors are likely to increase (in cases of high diversity) or decrease (in cases of low diversity) the relative value of the ‘cut-height’ that defines an approximate genus cutoff. This highlights the limitation of setting a 'one size fits all' dendrogram cutoff to distinguish between bacterial taxa and emphasizes that individual dendrograms and taxa must be analyzed on a case-by-case basis. Despite this limitation, our approach to set an approximate cutoff was highly effective toward identifying where multiple diverse bacterial genera were grouping and allowed us to rapidly identify members of those taxa with high confidence. Even when incorporating the additional step of approximating a genus cutoff, it is still significantly more efficient and cost effective than the current approaches in the field that use morphology or gene sequencing to guide strain selection. MALDI-MS/IDBac is a particularly useful technique when attempting to prioritize bacteria from environmental diversity plates, a common practice for bacterial natural product discovery labs. It should be noted that setting a dendrogram cut height is *not* necessary for this technique to be used, as the cut height acts only as a guideline to prioritize groupings that contain seeds.

### Identification of understudied genera in the dendrogram

Once the cutoff was verified to approximate genus level groupings, we assessed the ability of our method to identify isolates that belonged to understudied genera. A total of 22 isolates grouped with seeds of understudied genera. Of those 22 isolates, we were able to obtain 16S rRNA gene sequencing data from all of them and 19 accurately matched with the seed genus in their respective groupings (matches spanned five genera). Of the 19 isolates that matched correctly with their seeds, all of them had a parent node at or within the cut height of 6.5 in the dendrogram (86.3% success rate; Fig. 3).

For example, *Citrobacter* was chosen as a target understudied genus due to its average genome size estimated at 5.17 Mbp based on entries compiled in GTDB [53] and only a single reported natural product in peer-reviewed literature [54]. Therefore, identifying isolates within this genus represents a promising strategy toward uncovering novel natural product space. Two groupings of *Citrobacter* were identified at cut heights of 5.7 and 5.8. In the grouping at 5.7, three isolates matched with the seed *Citrobacter_braakii*_EB1I002A, while at 5.8 two isolates matched with the seed *Citrobacter_*sp_EB1K011. All five isolates were verified to be *Citrobacter* spp. via 16S rRNA gene sequencing analysis. Importantly, the *Citrobacter* seed EB1I002A that correlated with EBM012B were isolated from two different freshwater sponges (for further analysis on matches see Table S5 and supplemental discussion). This helped validate that the method was effective at grouping phylogenetically similar isolates from environmental diversity plates prepared from different samples. Similar results were observed in *Bosea* groupings (4.9: three isolates from two distinct samples; 6.3: three isolates from two distinct samples), *Hafnia* groupings (5.9: two isolates from one sample; 5.2: four isolates from two distinct samples; 3.1: one isolate from one sample), and *Variovorax* groupings (6.2: one isolate matching with seeds from two distinct seed samples). Similarly, the grouping of seed *Bosea*/Uncultured_LYD017 with isolates LYD003 and LYD005, among other examples, highlights the effectiveness of the method to distinguish between colonies isolated from the same sample (and even the same petri dish).

### Practicality of this approach to target bacterial taxa from the environment

Despite many challenges that remain toward employing MALDI-TOF MS to group environmental bacterial isolates, this study highlights significant advantages. First, the process described in this study requires less time and fewer resources when compared to more traditional gene sequencing-based analyses. The process of DNA extraction, polymerase chain reaction (PCR), gel electrophoresis, PCR cleanup and sequencing can take days for just 20-30 isolates and requires many consumables. With the MALDI approach, it is feasible to process up to 384 strains in roughly 4 hours when measuring time from the transfer of material onto a MALDI target plate with sterile toothpicks, to data visualization and analysis within IDBac [25]. Second, to identify specific taxa from a collection of bacterial isolates using sequencing-based approaches, researchers must sequence all isolates within their collection (high cost and time investment) or select isolates based on a target morphology (taxa often have overlapping or unknown variances in morphology). Third, MALDI-TOF MS provides more information than 16S rRNA gene sequencing, as an array of ionizable proteins drive these analyses versus just a single region of the genome. While whole genome sequencing is currently the most effective way to identify a bacterial isolate, it becomes cost and time prohibitive for large collections of bacteria. However, a few challenges remain toward the extensive application of this method, in addition to the challenges described earlier for instituting a dendrogram cut-height. For example, while many institutions maintain MALDI-TOF MS instruments, data collection accessibility may be limited in some research settings. Additionally, this approach requires genetically verified seed spectra of target taxa, which some laboratories may not possess. A potential solution for this issue is purchasing strains from accredited vendors, though this approach may become costly. A further and more comprehensive solution is the creation of an open-access and centralized protein MS repository with downloadable spectra, which our team is currently developing. Despite these limitations, MALDI-TOF MS/IDBac is ideal for processing a large volume of isolates when the downstream goal is to identify, group, prioritize, or dereplicate isolates. Development of a publicly available IDBac web platform to enhance these functions is currently underway.

## Conclusion

In the current study, a MALDI-TOF MS/IDBac pipeline was employed to rapidly mine a collection of environmental isolates for understudied bacterial genera. We demonstrated that inserting reference seeds into a dendrogram of samples provided ‘phylogenetic scale’ to similarity groupings and helped facilitate the identification process. This method is particularly useful when mining for bacteria that do not exhibit distinct morphological traits that could otherwise guide isolation efforts. This approach can save considerable sequencing costs if used to prioritize isolates from large collections of bacteria that have high probability of containing phylogenetic redundancy [25]. When coupled to high-throughput colony picking, this method has the potential to mine hundreds to thousands of environmental bacterial isolates for target taxa, adding a much-needed tool for researchers in chemical/microbial ecology and natural products discovery. Its effectiveness would also be amplified in the presence of a freely available database of bacterial reference spectra.

## Supplementary Material

SI_resub_Final_ycaf046

## Data Availability

MALDI-TOF MS data were deposited in MassIVE (https://doi.org/doi:10.25345/C5BG2HN4V, accession: MSV000094968). Partial 16S rRNA sequences of environmental seed and unknown bacteria used in this study were deposited in GenBank (Tables S1, S5, and S8).
