# Supplementary material for: Use of MALDI-TOF mass spectrometry and IDBac to mine for understudied bacterial genera from the environment
Source: ISME Commun. 2025 Mar 13;5(1):ycaf046. doi: 10.1093/ismeco/ycaf046 (PMC11962939; doi:10.1093/ismeco/ycaf046)
Supplement: SI_resub_Final_ycaf046 [file si_resub_final_ycaf046.pdf]

Use of MALDI-TOF mass spectrometry and IDBac to selectively mine for understudied bacterial genera from the environment.

Antonio Hernandez[τ,1,2]; Nyssa K. Krull[τ,1,2]; Brian T. Murphy[1,2,3*]

[1]Department of Pharmaceutical Sciences, College of Pharmacy, University of Illinois at Chicago, Chicago, IL, 60607, USA

[2]Center for Biomolecular Sciences, College of Pharmacy, University of Illinois at Chicago, Chicago, IL, 60607, USA

[3]Institute for Tuberculosis Research, College of Pharmacy, University of Illinois at Chicago, Chicago, IL 60612, United States

[τ] Authors contributed equally to this work.

[*] To whom correspondence should be addressed: btmurphy@uic.edu

Table of Contents

SUPPORTING INFORMATION (SI)

Table S1. Sample source and sample location of seed strains from well-studied (18 strains) genera and understudied genera (50 strains) 3

Table S2. Seed strains used in this study 6

Table S3. Well studied and understudied genera identified in the dendrogram with their respective parent nodes 7

Figure S1. Further inspection of three *Pseudomonas* groupings across the MALDI MS protein dendrogram 8

Figure S2. Examples of environmental isolates that grouped to seeds of understudied taxa at cut height of 6.5 or below, but *did not* match with seed genus via 16S rRNA gene sequencing 9

Figure S3. Examples of MALDI MS spectra (A) and a mirror plot of matched spectra between two Pseudomonas strains WIAJ06 and WIAJ08 (B) 10

Table S4. Adjusted Wallace coefficient for 4.0, 6.5, and 15.0 cut heights 12

Table S5. Sample information of understudied seed genera and their environmental isolate matches 13

Table S6. Table of collection expedition locations and isolate sample sources 14

Table S7. Bacterial growth media composition 16

Table S8. Additional NCBI accension numbers from well-studied genera that grouped with seed strains 18

Supplemental discussion 19

References 20

Table S1. Sample source and sample location of seed strains from well-studied genera (18 strains, shaded) and understudied genera (50 strains).

Isolate legend: Iceland = Icelandic seeds, AIS = Apostle Islands seeds, MLL = Murphy lab seeds, MS = Multi-state seeds, JPL = Jet Propulsion Lab seeds. NRRL: Denotes strain acquired through the NRRL Agricultural Research Service Culture Collection (https://nrrl.ncaur.usda.gov/).

Genus	Species	Isolate ID	Sample source	Sample location (coordinates)	NCBI Accession number
Pseudomonas	*protegens*	MS - WIAJ01	Freshwater macroalgae 1	44.086796, -88.710487	PP723248.1
	protegens	MS - WIAJ02	Freshwater macroalgae 1	44.086796, -88.710487	PP723247.1
	protegens	MS - WIAJ03	Freshwater macroalgae 1	44.086796, -88.710487	PP723246.1
	protegens	MS - WIAJ06	Freshwater macroalgae 1	44.086796, -88.710487	PP724693.1
	protegens	MS - WIAJ08	Freshwater macroalgae 1	44.086796, -88.710487	PP724694.1
	protegens	MS - WIAJ12	Freshwater macroalgae 1	44.086796, -88.710487	PP723322.1
	protegens	MS - WIAJ13	Freshwater macroalgae 1	44.086796, -88.710487	PP723325.1
	protegens	MS - WIAJ14	Freshwater macroalgae 1	44.086796, -88.710487	PP723327.1
	protegens	MS - WIAJ15	Freshwater macroalgae 1	44.086796, -88.710487	PP723326.1
	glycinae	MS - EB2J004	Freshwater sponge 2	46.981932, -123.412435	PP665745.1
	sp.	MS - WTHA10	Freshwater sediment LHS	43.579528, -103.442718	PP723333.1
	sp.	MS - WTHF07	Freshwater sediment RHS	43.579528, -103.442718	PP723332.1
	sp.	MS - WTHI02	Freshwater sediment LHS	43.579528, -103.442718	PP723342.1
Hymenobacter	sp.	MS - LYG005A	Lichen	41.936143, -87.764957	PP723064.1
Methylobacterium	sp.	MS - LYG010C	Lichen	41.936143, -87.764957	PP723074.1
	sp.	MS - LYG013A	Lichen	41.936143, -87.764957	PP723073.1
	sp.	MS - LYH001B	Lichen	41.936143, -87.764957	PP723110.1
Erwinia	*billingiae*	MS - MS2C06Y	Macroalgae	41.936143, -87.764957	PP723171.1
Mycolicibacterium	sp.	Iceland - 135A-30	Marine sediment	65.73357, -18.170398	MK143199
Blastococcus	sp.	Iceland - 137A-25	Marine sponge	65.484244, -22.391858	MK163426

Janibacter	sp.	Iceland - 137A-17	Marine sponge	65.484244, -22.391858	MK143181
Friedmanniella	sp.	Iceland - 114A-2	Marine sponge	66.0278306, -18.4062778	MK143176
Marmoricola	sp.	Iceland - 120A-11	Sand	65.721916, -16.792269	MK143135
Oerskovia	sp.	Iceland - 116H-18	Freshwater sediment	66.098072, -16.9298790	MK163385
Kineosporiaceae (family)	N/A	Iceland - 114E-7	Marine water	66.0278306, -18.4062778	MK143184
Arthrobacter	sp.	Iceland - 117A-1	Freshwater sediment	66.076302, -16.6882450	MK143208
	sp.	Iceland - 117G-16	Freshwater sediment	66.076302, -16.6882450	MK143115
	sp.	Iceland - 117G-22	Freshwater sediment	66.076302, -16.6882450	MK143163
	sp.	Iceland - 117G-24	Freshwater sediment	66.076302, -16.6882450	MK163387
	sp.	Iceland - 117G-33	Freshwater sediment	66.076302, -16.6882450	MK143161
	sp.	Iceland - 117G-35	Freshwater sediment	66.076302, -16.6882450	MK163388
	sp.	Iceland - 119A-10	Freshwater mud	65.72176, -16.788817	MK168025
Williamsia	sp.	Iceland - 137A-20	Marine sponge	65.484244, -22.391858	MK143189
Maribacter	sp.	Iceland - 118B-36	Marine algae	66.076305, -16.68827	MK143311
	sp.	Iceland - 138H-4	Marine sponge	65.512811, -22.304967	MK143373
Aquimarina	sp.	Iceland - 138H-63	Marine sponge	65.512811, -22.304967	MK143212
	sp.	Iceland - 138H-88	Marine sponge	65.512811, -22.304967	MK143215
Dyadobacter	sp.	Iceland - 129A-45	Freshwater sediment	65.638311, -16.910157	MK143271
Flavobacterium	sp.	Iceland - 138H-11	Marine sponge	65.512811, -22.304967	MK143220
Bosea	sp.	Iceland - 128A-35	Freshwater sponge	65.640207, -16.844106	MK163411
Variovorax	sp.	Iceland - 119A-16	Freshwater mud	65.72176, -16.788817	MK163397
Cohnella	sp.	Iceland - 130A-12	Freshwater sediment	65.579718, -16.951147	MK143287
Chryseobacterium	sp.	AIS - SP168	Freshwater sponge	46.909167, -90.624167	PP766728.1
Serratia	sp.	AIS - SP418	Freshwater sponge	46.882222, -90.675556	PP766729.1

Actinomycetospora	*chiangmaiensis*	MLL - Z002	Marine snail (surface swab)	32.866516, -117.2507033	PP779713.1
Mycolicibacterium	sp.	MS - LYG016B	Lichen	43.579528, -103.442718	PP723079.1
Chitinophaga	sp.	MS - AC21	Soil	43.579528, -103.442718	-
Serratia	sp.	MS - MADAA01	Marine sponge	42.375804, -70.995221	PP724689.1
	sp.	MS - MADAA04	Marine sponge	42.375804, -70.995221	PP719691.1
	sp.	MS - EB1H004B	Freshwater sponge 1	46.981932, -123.412435	PP719631.1
	fonticola	MS - EB1H004A	Freshwater sponge 1	46.981932, -123.412435	PP719819.1
Citrobacter	*braakii*	MS - EB1I002A	Freshwater sponge 1	46.981932, -123.412435	PP719632.1
	sp.	MS - EB1K011	Freshwater sponge 1	46.981932, -123.412435	PP723009.1
Hafnia	*paralvei*	MS - EB1K020	Freshwater sponge 1	46.981932, -123.412435	PP719656.1
	sp.	MS - EB1K016	Freshwater sponge 1	46.981932, -123.412435	PP724702.1
Bosea	sp.	MS - LYD010	Moss 1	41.936143, -87.764957	PP723059.1
	sp.	MS - LYD011	Moss 1	41.936143, -87.764957	PP724690.1
	sp.	MS - LYD012	Moss 1	41.936143, -87.764957	PP724691.1
	sp.	MS - LYD014	Moss 1	41.936143, -87.764957	PP723062.1
Variovorax	sp.	MS - LYG003A	Lichen	41.936143, -87.764957	PP719678.1
Kocuria	*rosea*	JPL - MSL_359	Cleanroom	-	JAMAXV000000000
	palustris	JPL - MER_TA_14	Cleanroom	-	JAMAVY000000000
Ralstonia	*pickettii*	JPL - MSL_336.2	Cleanroom	-	JAMAXT000000000
Cupriavidus	*pauculus*	JPL - MSL_348	Cleanroom	-	JAMAXU000000000
Acidobacteriaceae (family)	sp.	Acido_24188	-	-	ATWD00000000
Acidobacteriaceae (family)	sp.	Acido_24295	-	-	FJ870383
Bryobacterales (family)	sp.	Acido_24297	-	-	ARMF00000000
Variovorax	*paradoxus*	B-1908	-	-	NRRL

Table S2. Seed strains used in this study. In total, 50 seed isolates spanned 5 phyla and 24 genera. Average genome size was calculated by downloading the GTDB database and averaging the genome size of each seed genus. NPAtlas and literature searches using the SciFinder database were used to determine the number of known compounds published for each genus.

α: *Serratia* spp., *Flavobacterium* sp., *and Chitinophaga* sp. are exceptions to target genera having fewer than 20 published NPs

Phylum	Genus	Average Genus Genome Size	# of known compounds – reported (05/2024)	Reference(s) for reported compounds
Actinomycetota	*Mycolicibacterium*	6.22 Mbp	2	(1)
	Blastococcus	4.34 Mbp	0	-
	Janibacter	3.11 Mbp	17	(2)
	Friedmanniella	4.28 Mbp	0	-
	Marmoricola	3.61 Mbp	0	-
	Oerskovia	4.33 Mbp	4	(3)
	Arthrobacter	4.26 Mbp	12	(4–7)
	Williamsia	5.40 Mbp	3	(8)
	Actinomycetospora	5.99 Mbp	3	(9,10)
	Kocuria	3.07 Mbp	5	(11)
Bacteroidota	*Maribacter*	4.10 Mbp	0	-
	Aquimarina	5.22 Mbp	10	-
	Dyadobacter	6.96 Mbp	0	-
	Flavobacterium$^{\alpha}$	3.41 Mbp	40	-
	Chryseobacterium	4.58 Mbp	13	(12)
	Chitinophaga	7.32 Mbp	23	(13,14)
Bacillota	*Cohnella*	6.48 Mbp	0	-
Psuedomonadota	*Serratia*$^{\alpha}$	5.28 Mbp	46	-
	Citrobacter	5.16 Mbp	1	-
	Hafnia	4.73 Mbp	0	-
	Bosea	5.28 Mbp	0	-
	Variovorax	6.84 Mbp	11	(15)
	Ralstonia	5.54 Mbp	16	-
	Cupriavidus	6.56 Mbp	1	-
Acidobacteriota	-	-	0	-
			0	-

Table S3. Well studied and understudied genera identified in the dendrogram with their respective parent nodes.

Seed genus	Parent node height	Total non-seed isolates in grouping	Total isolates with 16S rRNA gene sequencing data	Number of 16S rRNA verified genus-level matches
Pseudomonas	6.5	1	1	1
	5.9	7	6	6
	4.7	0	0	0
Methylobacterium	6.4	4	4	4
	5.7	3	2	2
	3.2	1	1	0
Hymenobacter	5.1	3	2	2
Erwinia	3.2	1	1	1
Bosea	4.9	3	3	3
	6.3	3	3	3
	6.3	0	0	0
	6.4	2	2	1
Citrobacter	5.8	2	2	2
	5.7	3	3	3
Hafnia	3.1	1	1	1
	5.2	4	4	4
	5.9	2	2	2
Variovorax	6.2	1	1	1
Kocuria	6.3	1	1	0
Marmoricola	6.4	1	1	0
Serratia	5.7	1	1	0
total		43	40	35

Figure S1. Further inspection of three *Pseudomonas* groupings across the MALDI MS protein dendrogram. A) Three groupings of *Pseudomonas* spp. in the dendrogram with isolates at cut heights 6.5, 5.9, and 4.7. B) A phylogenetic tree of the same three groupings of *Pseudomonas* spp. with *Bacillus* spp. as an outgroup based on partial 16S rRNA gene sequencing analysis. *Bacillus* spp. can be found under the following NCBI accession numbers: PP779731.1, PP779733.1, PP779734.1, PP780015.1, PP816724.1, and PP816725.1.

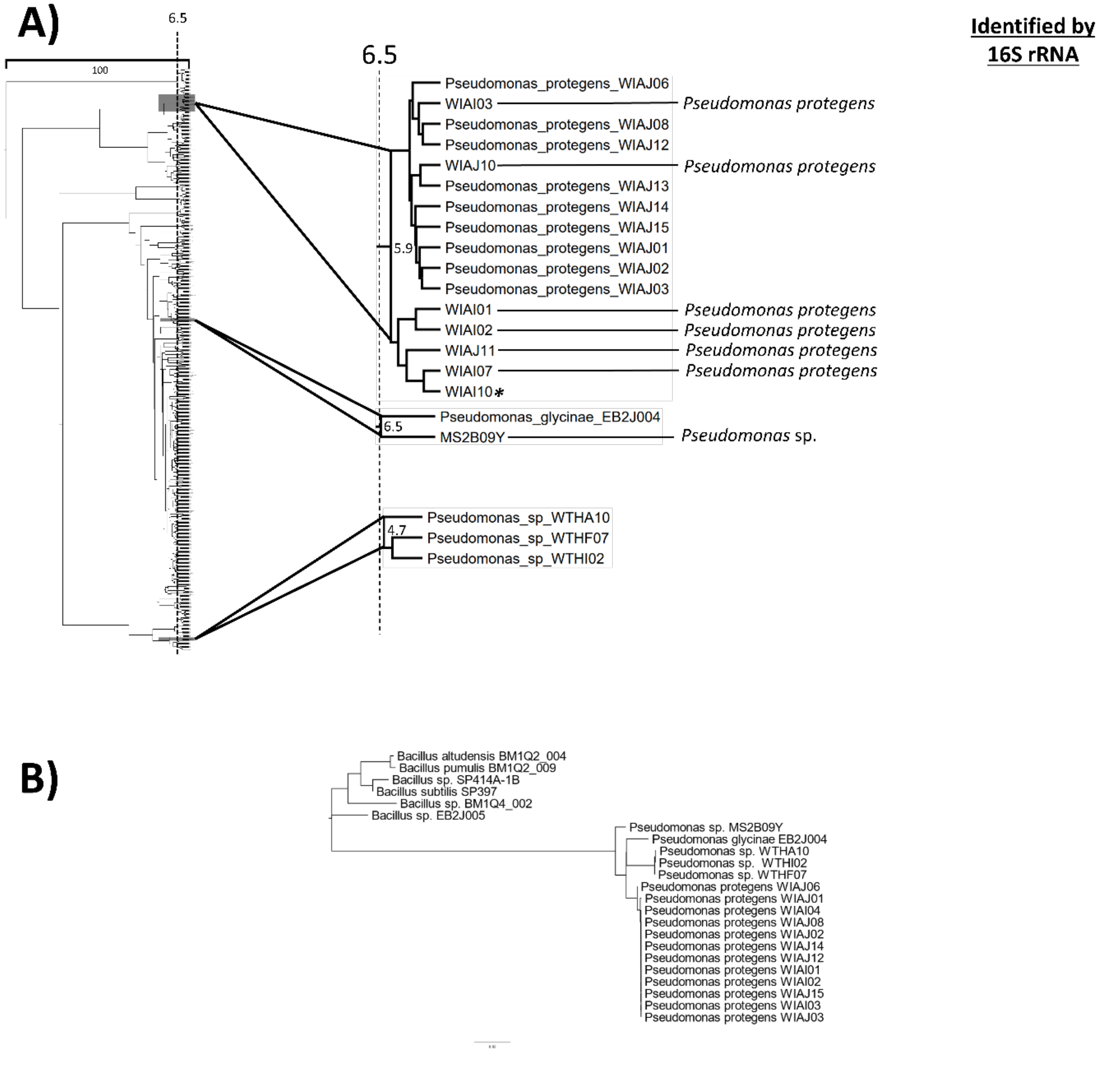

*16S rRNA gene sequencing analysis was inconclusive due to cryostock contamination, therefore a reliable isolate ID was not possible.

Creation of Phylogenetic tree of *Pseudomonas* spp. based on 16S rRNA gene sequencing.

The phylogenetic tree in figure S1B was generated using Geneious Prime software version 2023.2.1. Geneious Tree Builder was selected, and the following parameters were used to create the phylogenetic tree: multiple alignment of 16S rRNA sequences, Tamura-Nei genetic distance model, neighbor-joining tree build method.

Figure S2. Examples of environmental isolates that grouped to seeds of understudied taxa at cut height of 6.5 or below, but *did not* match with seed genus via 16S rRNA gene sequencing.

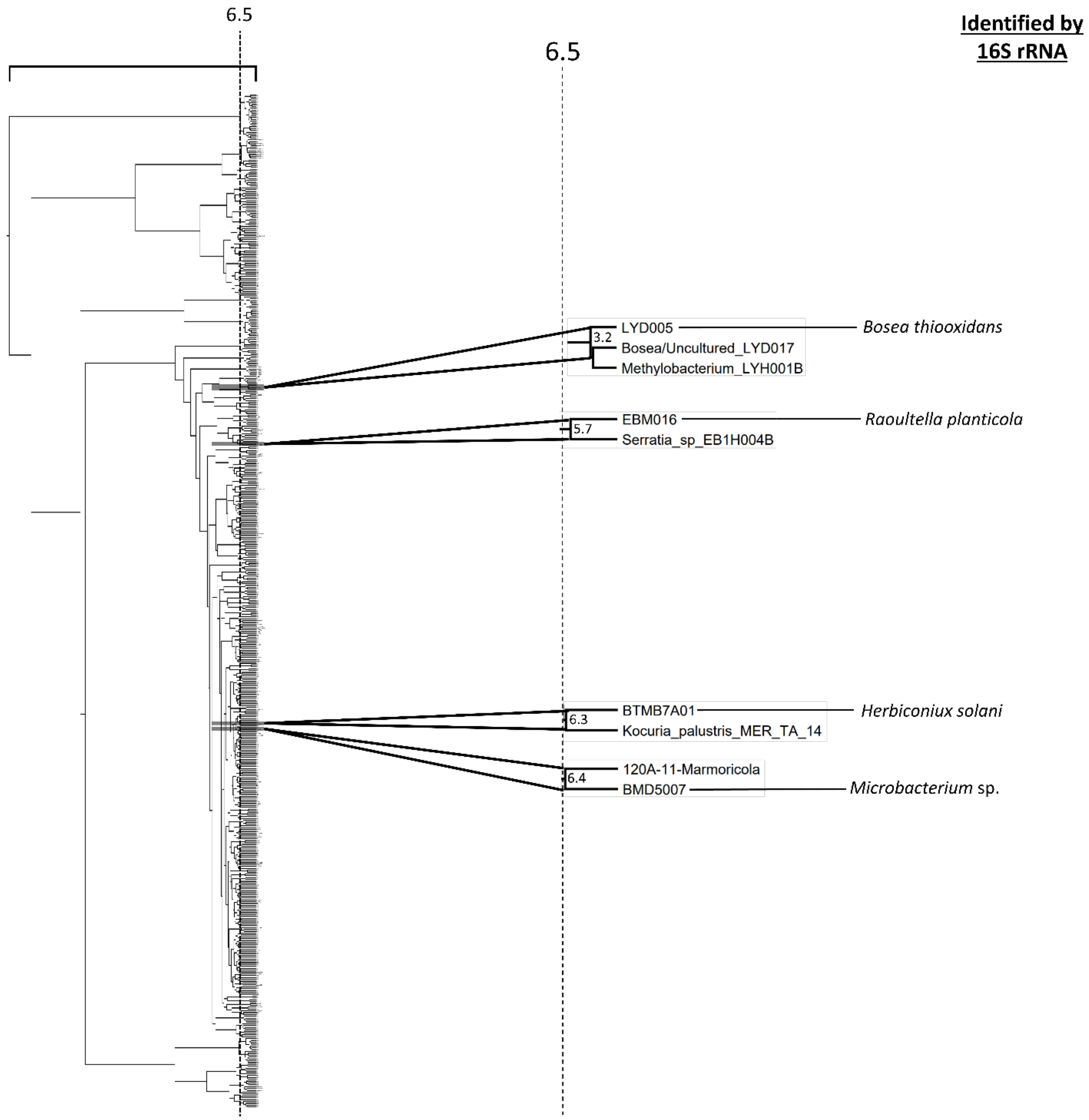

Figure S3. Examples of MALDI MS spectra (A) and a mirror plot of matched spectra between two *Pseudomonas* strains WIAJ06 and WIAJ08 (B).

A. Example of spectra used in this study.
(Top: Pseudomonas WIAJ08, Bottom: Pseudomonas WIAJ06)

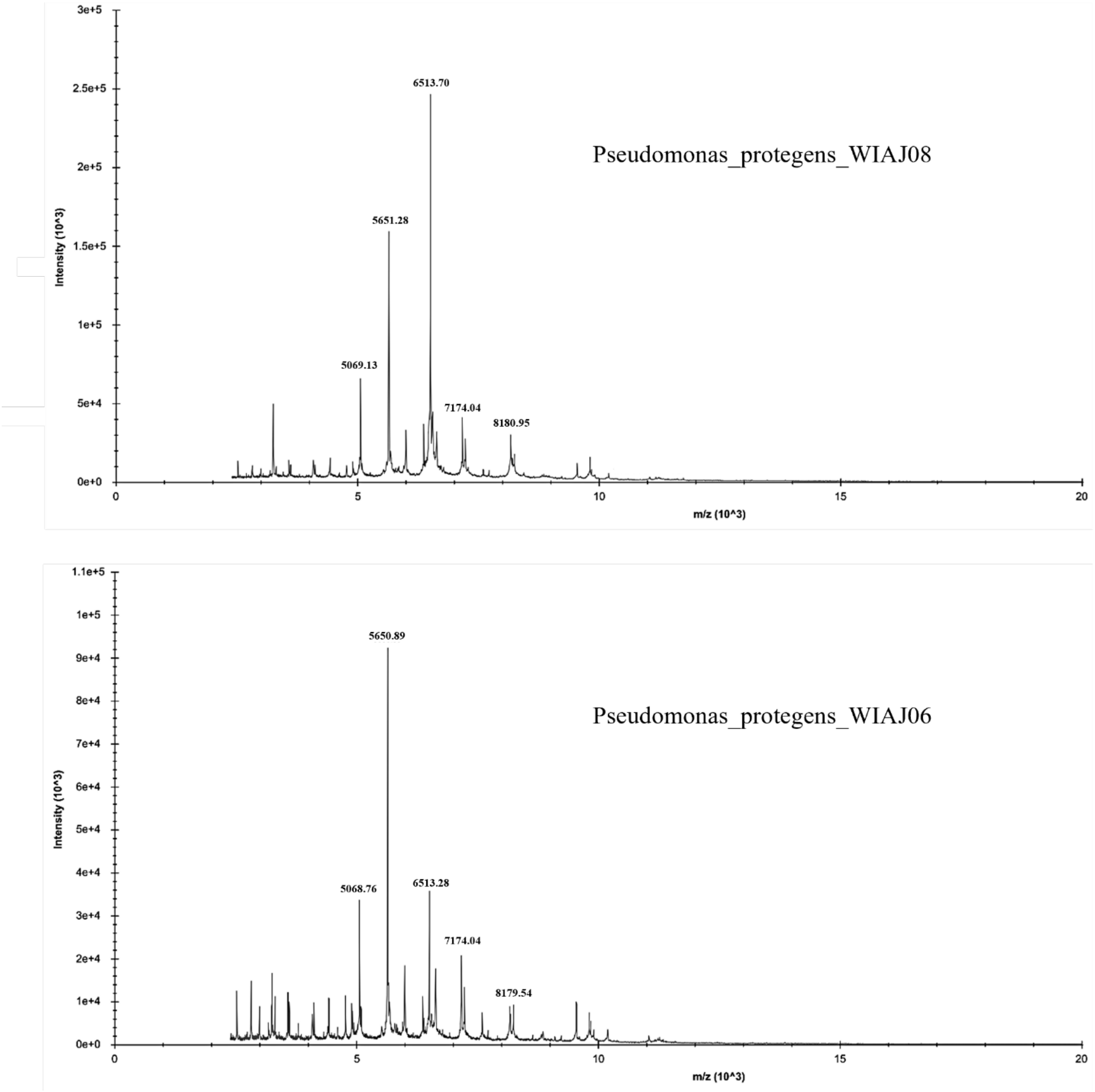

B. Mirror plot of spectra from *Pseudomonas* WIAJ08 and WIAJ06. Blue denotes peak matches, red denotes peaks with no match.
(Top: *Pseudomonas* WIAJ08, Bottom: *Pseudomonas* WIAJ06)

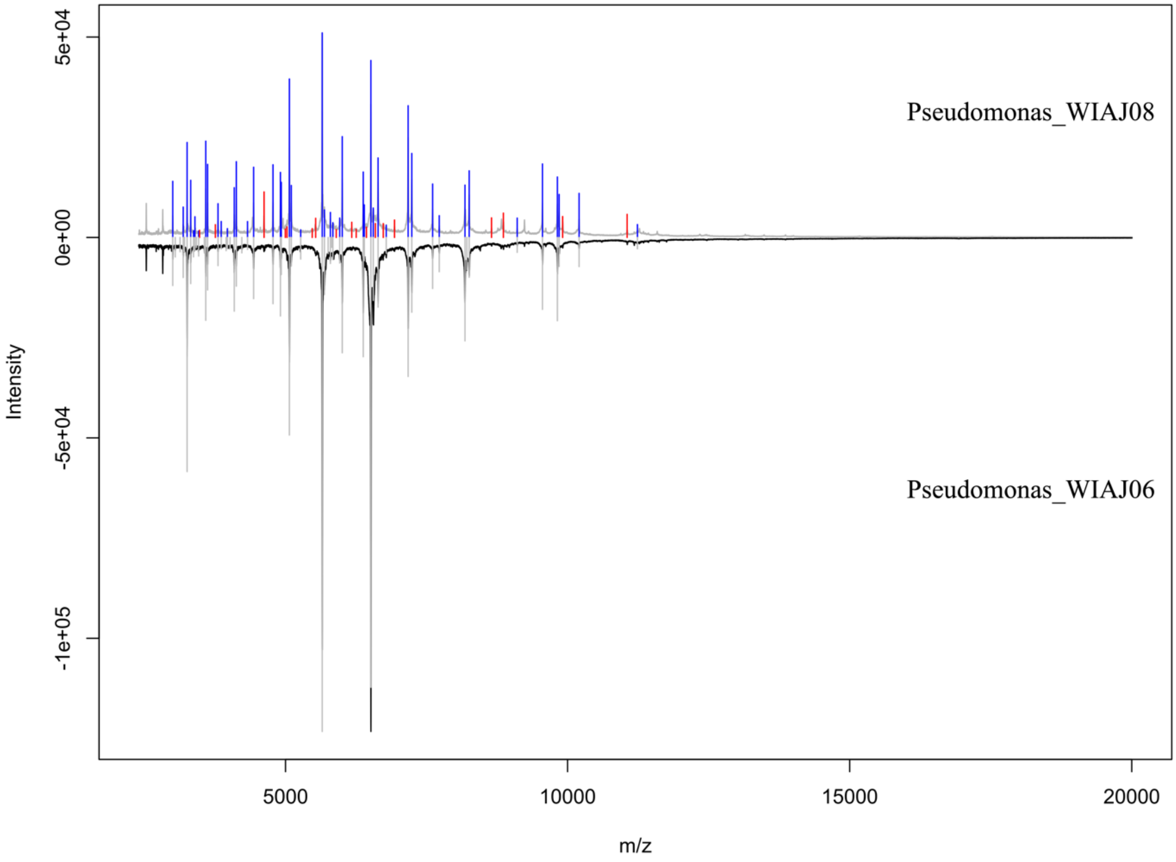

Table S4. Adjusted Wallace coefficient for 4.0, 6.5, and 15.0 cut heights. The adjusted Wallace coefficient is directional and provides a measure of how similar clustering results are within a dataset (95% confidence intervals are listed in parenthesis). Here, the 'Likelihood…' column shows the probability of members of the same genus to group in a single cluster at three distinct cut heights. A cut at 6.5 shows a 93.5 percent chance that strains within a cluster are members of the same genus. A 4.0 cut height increases this to a 97 percent chance, though the improvement excludes members of the same genus that are captured at a 6.5 cut.

**Cluster cut height**	**Likelihood entities in a cluster are of the same genus**
4.0	0.970<br>(0.948-0.991)
6.5	0.935<br>(0.908-0.963)
15.0	0.050<br>(0.039-0.062)

Table S5. Sample information of understudied seed genera and their environmental isolate matches.

Cut height	Genus	Species	Isolate ID	Source organism	NCBI Accession number
6.4	*Bosea*	sp.	LYB009	Moss 1	PP723013.1
	Pseudomonas	*protegens*	BTMB8D02	Moss	PP779730.1
6.3	[S]*Bosea*	sp.	LYD010	Moss 1	PP723059.1
	[S]*Bosea*	sp.	LYD014	Moss 1	PP723062.1
6.3	*Bosea*	sp.	LYH003B	Lichen	PP723101.1
	Bosea	*thiooxidans*	LYD005	Moss 1	PP723055.1
	Bosea	sp.	LYD003	Moss 1	PP723054.1
	[S]*Bosea*	sp.	LYD017	Moss 1	PP723063.1
	[S]*Methylobacterium*	sp.	LYH001B	Lichen	PP723110.1
4.9	*Bosea*	sp.	LYC002	Moss 2	PP724667.1
	Bosea	sp.	LYC001	Moss 2	PP722973.1
	Bosea	*massiliensis*	LYB013	Moss 1	PP724666.1
	[S]*Bosea*	sp.	LYD012	Moss 1	PP724691.1
	[S]*Bosea*	sp.	LYD011	Moss 1	PP724690.1
5.8	*Citrobacter*	*tructae*	EB1K003	Fresh Water Sponge 1	PP719817.1
	Citrobacter	*freundii*	EB1K015	Fresh Water Sponge 1	PP735251.1
	[S]*Citrobacter*	sp.	EB1K011	Fresh Water Sponge 1	PP723009.1
5.7	*Citrobacter*	sp.	EB1H001B	Fresh Water Sponge 1	PP719699.1
	Citrobacter	sp.	EB1H002B	Fresh Water Sponge 1	PP719698.1
	Citrobacter	*freundii*	EBM012B	Fresh Water Sponge 2	PP723014.1
	[S]*Citrobacter*	*braakii*	EB1I002A	Fresh Water Sponge 1	PP719632.1
5.9	*Hafnia*	sp.	EB1K012	Fresh Water Sponge 1	PP722975.1
	Hafnia	sp.	EB1K002	Fresh Water Sponge 1	PP719820.1
	[S]*Hafnia*	*paralvei*	EB1K020	Fresh Water Sponge 1	PP719656.1
5.2	*Hafnia*	sp.	EBM010A	Fresh Water Sponge 2	PP723008.1
	Hafnia	*alvei*	EB1K007	Fresh Water Sponge 1	PP722682.1
	Hafnia	*alvei*	EB1K005	Fresh Water Sponge 1	PP722681.1
	Hafnia	*alvei*	EB1K010	Fresh Water Sponge 1	PP719864.1
3.1	*Hafnia*	sp.	EB1K018	Fresh Water Sponge 1	PP724703.1
	[S]*Hafnia*	sp.	EB1K016	Fresh Water Sponge 1	PP724702.1
6.2	*Variovorax*	sp.	MS2C04P	Macroalgae	PP723172.1
	[S]*Variovorax*	sp.	LYG003A	Lichen	PP719678.1
	[S]*Variovorax*	sp.	B-1908	-	NRRL
6.7	*Kocuria*	*rhizophila*	BM4AG004	Alkaline pond water	PP756383.1
	Herbiconiux	*solani*	BTMB7A01	Macroalgae	PP756384.1
	[S]*Kocuria*	*palustris*	MER_TA_14	Cleanroom	JAMAVY000000000

NRRL: Denotes cell material acquired through the NRRL Agricultural Research Service Culture Collection (https://nrrl.ncaur.usda.gov/) through collaboration with Dr. William Metcalf at University of Illinois Urbana Champaign. *Note: no live cell material was transferred to UIC, in accordance with existing MTAs.*

[S]: Denotes seed strain.

Table S6. Table of collection expedition locations and isolate sample source.

Isolate ID prefix	Sample Source	Cultivation media	Sample collection location/ coordinates
BM2AF	Freshwater macroalgae 1	629	'Big Marsh' Chicago, IL 41.687062, -87.574037
BM2AG	Freshwater macroalgae 2	M31	'Big Marsh' Chicago, IL 41.687062, -87.574037
BM4	Alkaline pond water	VL55	'Big Marsh' Chicago, IL 41.687062, -87.574037
BM5	Alkaline pond sediment 1	VL55	'Big Marsh' Chicago, IL 41.687062, -87.574037
BM6	Alkaline pond sediment 2	VL55	'Big Marsh' Chicago, IL 41.687062, -87.574037
BTMB1	Moss 1	629	Winthrop, MA 42.361515, -70.974625
BTMB3	Moss 2	M31	Winthrop, MA 42.361515, -70.974625
BTMB4	Marine sponge	M31	Winthrop, MA 42.375804, -70.995221
BTMB5	Marine sediment	629	Winthrop, MA 42.375804, -70.995221
BTMB6	Marine macroalgae 1	629	Winthrop, MA 42.375804, -70.995221
BTMB7	Marine macroalgae 2	M31	Winthrop, MA 42.375804, -70.995221
BTMB8	Moss 3	629	Winthrop, MA 42.375804, -70.995221
EB1	Freshwater sponge 1	A1	Chehalis River, WA 46.981932, -123.412435
EB2	Freshwater sponge 2	A1	Chehalis River, WA 46.981932, -123.412435
EBM	Freshwater sponge 2	A1	Chehalis River, WA 46.981932, -123.412435
LYA	Soil	M31	Chicago, IL 41.936143, -87.764957
LYB	Moss 1	629	Chicago, IL 41.936143, -87.764957
LYC	Moss 2	M31	Chicago, IL 41.936143, -87.764957
LYD	Moss 1	M31	Chicago, IL 41.936143, -87.764957
LYG	Lichen	2670	Chicago, IL 41.936143, -87.764957
LYH	Lichen	2670	Chicago, IL 41.936143, -87.764957
LYZ	Moss 1	2670	Chicago, IL 41.936143, -87.764957
MS2A	Moss on a rock	M31	Ellison Bay, WI 45.215663, -87.039573
MS2B	Moss on a tree	629	Ellison Bay, WI 45.215663, -87.039573
MS2C	Moss on decaying log	M31	Ellison Bay, WI 45.215663, -87.039573
MS2D	Decaying log/detritus	629	Ellison Bay, WI 45.215663, -87.039573
MS2E	Lichen	629	Ellison Bay, WI 45.215663, -87.039573
MS2F	Freshwater sediment	M31	Ellison Bay, WI 45.215663, -87.039573
MS2G	Freshwater macroalgae	M31	Ellison Bay, WI 45.215663, -87.039573
MA3	Marine water column	VL55	Winthrop, MA 42.375804, -70.995221
MADAA	Marine sponge	629	Winthrop, MA 42.375804, -70.995221
OS2	Homogenized cockroach	A1	Chicago, IL 41.940412, -87.702483
OS3	Moss	629	Ann Arbor, MI 42.282338, -83.748201
WIAB	Freshwater sediment	M31	Lake Butte Des Morts, WI 44.086796, -88.710487
WIAD	Freshwater macroalgae 1	629	Lake Butte Des Morts, WI 44.086796, -88.710487
WIAE	Freshwater macroalgae 2	629	Lake Butte Des Morts, WI 44.086796, -88.710487
WIAG	Freshwater sediment with water column	M31	Lake Butte Des Morts, WI 44.086796, -88.710487
WIAI	Freshwater macroalgae 3	629	Lake Butte Des Morts, WI 44.086796, -88.710487
WIAJ	Freshwater macroalgae 4	629	Lake Butte Des Morts, WI 44.086796, -88.710487
WIXX	Freshwater macroalgae 5	M31	Lake Butte Des Morts, WI 44.086796, -88.710487
WTHA	Cave lake sediment 1	A1	Wind Cave National Park 43.579528, -103.442718
WYHB	Calcite Dump Filtered	A1	Wind Cave National Park 43.579528, -103.442718
WTHC	Sediment RHS 1	A1	Wind Cave National Park 43.579528, -103.442718

WTHD	Cave lake water 1	A1	Wind Cave National Park 43.579528, -103.442718
WTHE	Cave lake water 2	A1	Wind Cave National Park 43.579528, -103.442718
WTHF	Sediment RHS 2	M31	Wind Cave National Park 43.579528, -103.442718
WTHG	Sediment LHS 2	M31	Wind Cave National Park 43.579528, -103.442718
WTHH	Cave lake water 2	M31	Wind Cave National Park 43.579528, -103.442718
WTHI	Sediment LHS 1	629	Wind Cave National Park 43.579528, -103.442718
WTHJ	Cave lake water 1	629	Wind Cave National Park 43.579528, -103.442718
WTHK	Cave lake water 2	629	Wind Cave National Park 43.579528, -103.442718
WTHL	Sediment RHS 3	629	Wind Cave National Park 43.579528, -103.442718
WTHO	Sediment RHS 4	A1	Wind Cave National Park 43.579528, -103.442718

Table S7. Bacterial growth media composition.

Media type	Ingredient	Weight/vol (g/L)
A1	Soluble Starch	10 g
	Yeast extract	4 g
	Peptone	2 g
	Sterilized dH_2O	1000 mL
M31<br><br>**Adapted from Ivanova et al., 2016** (16)	KH2PO4	0.1 g
	Hutner's basal salts:<br>Nitrilotriacetic acid (NTA), MgSO4. 7H20, CaCl2, 2H2O, (NH4) MoO7O24. 4H2O, FeSO4 . 7H2O<br><br>"Metal 44":<br>Na-EDTA, ZnSO4 . 7H2O, FeSO4 . 7H2O, MnSO4 . H2O, CuSO4 . 5H2O, Co(NO3)2 . 6H2O, Na2B4O7 . 10H2O, Distilled water	20 mL
	N-acetylglucosamine	1.0 g
	Peptone	0.1 g
	Yeast extract	0.1 g
	Gellan gum	8.00 g
	Agar	8.00
	dH_2O	980 mL
629<br><br>**As described by Leibniz Institute DSMZ-German Collection of Microorganisms and Cell Cultures.**	Peptone	5.0 g
	Yeast extract	0.5 g
	Hutner's basal salts:<br>Nitrilotriacetic acid (NTA), MgSO4. 7H20, CaCl2. 2H2O, (NH4) MoO7O24. 4H2O, FeSO4. 7H2O<br><br>"Metal 44":<br>Na-EDTA, ZnSO4. 7H2O, FeSO4. 7H2O, MnSO4. H2O, CuSO4. 5H2O, Co (NO3)2. 6H2O, Na2B4O7. 10H2O, Distilled water	20.0 mL
	Staley's vitamins:<br>Vitamin B12, Biotin, Thiamine-HCl x 2 H2O, Ca-pantothenate, Folic acid, Riboflavin, Nicotinamide & Distilled water	10.0 mL
	Gellan gum	8.00 g
	Agar	8.00
	Distilled water	970.0 mL

Media type	Ingredient	Weight/vol (g/L)
2670 Singulosphaera Medium<br><br>**As described by American Type Culture Collection.**	N-acetylglucosamine	1.00 g
	Peptone	0.10 g
	Yeast Extract	0.10 g
	KH2PO4	0.10 g
	MgSO4. 7H20	0.05 g
	CaCl2. 2H2O	0.01 g
	Hutner's basal salts:	20.00 mL

	Nitrilotriacetic acid (NTA), MgSO4. 7H20, CaCl2. 2H2O, (NH4) MoO7O24 . 4H2O, FeSO4. 7H2O<br><br>“Metal 44”:<br>Na-EDTA, ZnSO4. 7H2O, FeSO4. 7H2O, MnSO4. H2O, CuSO4. 5H2O, Co (NO3)2. 6H2O, Na2B4O7, 10H2O, Distilled water	
	Gellan gum	8.00 g
	Agar	8.00
	dH_2O	1000 mL
VL55<br><br>**As described by Leibniz Institute DSMZ-German Collection of Microorganisms and Cell Cultures.** (**https://www.dsmz.de/microorganisms/medium/pdf/DSMZ_Medium1266.pdf**)	MES (2-Morpholinoethanesulfonic acid)	3.90 g
	20 mM MgSO4 x 7 H2O	20.00 mL
	30 mM CaCl2 x 2 H2O	20.00 mL
	20 mM (NH4)2HPO4	20.00 mL
	Selenite-tungstate solution:<br>NaOH, Na2SeO3 x 5 H2O, Na2WO4 x 2 H2O, & Distilled water	2.00 mL
	Trace element solution SL10:<br>HCl (25%; 7.7 M), FeCl2 x 4 H2O, ZnCl2, MnCl2 x 4 H2O, H3BO3, CoCl2 x 6 H2O, CuCl2 x 2 H2O, NiCl2 x 6 H2O, Na2MoO4 x 2 H2O, & Distilled water	2.00 mL
	Vitamin solution:<br>Vitamin B12, 4-aminobenzoate, Biotin, Nicotinic acid, Hemicalcium D-(+)- pantothenate, Pyridoxamine-HCl, Thiamine-HCl x 2 H2O, D,L-6,8-thioctic acid, Riboflavin, Folic acid, & Distilled water	1.50 mL
	0.2M Glucose	10.00 mL
	Gellan gum	8.00 g
	dH_2O	900 mL

Table S8. Additional NCBI accension numbers from well-studied genera that grouped with seed strains.

Genus	Species	Isolate ID	NCBI Accession number
Pseudomonas	*protegens*	MS - WIAJ11	PP756483.1
	protegens	MS - WIAI01	PP723213.1
	protegens	MS - WIAI02	PP724692.1
	protegens	MS - WIAI03	PP723222.1
	protegens	MS - WIAJ10	PP756492.1
	protegens	MS - WIAI07	PP756328.1
	sp.	MS - MS2B09Y	PP723133.1
Methylobacterium	sp.	MS - LYG017A	PP723081.1
	sp.	MS - LYH006	PP724668.1
	sp.	MS - LYB009A	PP756493.1
	sp.	MS - LYG009A	PP723013.1
Hymenobacter	sp.	MS - LYG014A	PP756498.1
Erwinia	billingiae	MS - MS2B04Y	PP723134.1

Supplemental Discussion.

Identification of understudied genera in the dendrogram.
Three groupings of *Hafnia* at cut heights of 5.9, 5.2, and 3.1; the average genome size of *Hafnia* was calculated to be 4.75 Mbp based on entries compiled in GTDB and this genus contains no reported natural products in the database NPAtlas and after review of peer-reviewed literature. We verified 7 isolates in these clusters to be *Hafnia* spp. via 16S rRNA gene sequencing analysis.

A grouping of *Variovorax* at cut height 6.2; the average genome size of *Variovorax* was calculated to be 7.06 Mbp based on entries compiled in GTDB and this genus contains eleven reported natural products in the database NPAtlas and after review of peer-reviewed literature. We verified one isolate in this cluster to be *Variovorax* spp. via 16S rRNA gene sequencing analysis. *Variovorax* seeds that correlated with the environmental *Variovorax* isolate were from different environmental samples.

There was a grouping between a *Kocuria* seed (*Kocuria_palustris*_MER_TA_14) and an isolate from another order (*Herbiconius solani*) at 6.3 cut height, however, in the node directly above the cut 6.3 there was a cut at 6.7 which correlated the same seed with a *Kocuria rhizophila* isolate. The average genome size of *Kocuria* was calculated to be 3.05 Mbp based on entries compiled in GTDB and this genus contains five reported natural products in the database NPAtlas and literature review. We verified 1 isolate in this cluster to be *Kocuria* sp. via 16S rRNA gene sequencing analysis. The *Kocuria* seed MER_TA_14 that correlated with the environmental *Kocuria* isolate were different environmental samples.

Three groupings of *Bosea* at cut height 6.4, 6.3, and 4.9; the average genome size of *Bosea* was calculated to be 5.70 Mbp based on entries compiled in GTDB and this genus contains no reported natural products in the database NPAtlas and after review of peer-reviewed literature. We verified 7 isolates in these clusters to be *Bosea* spp. via 16S rRNA gene sequencing analysis.

Interestingly, we observed a *Methylobacterium*_LYH001B grouping within a *Bosea* spp. cluster in one portion of the dendrogram. In previous studies, high 16S rRNA gene similarity was observed between these two genera (17, 18, 19). These data paired with the high resolution that MALDI-TOF MS and IDBac provides, suggests that *Bosea* and *Methylobacterium* spp. may be more closely related than the current taxonomic assignment implies. Ultimately, future studies are required to better understand the what drives the MALDI-TOF MS grouping and whether unexpected matches can be explained beyond contamination, or incorrect taxonomic designation.

References

1. Peña-Ortiz L, Graça AP, Guo H, Braga D, Köllner TG, Regestein L, et al. Structure elucidation of the redox cofactor mycofactocin reveals oligo-glycosylation by MftF. Chem Sci. 2020; 11:5182–5190.
2. Ding W, Li Y, Tian X, Xiao Z, Li R, Zhang S, et al. Investigation on metabolites in structure and biosynthesis from the deep-sea sediment-derived Actinomycete *Janibacter* sp. SCSIO 52865. Molecules. 2023; 28:2133.
3. Yamada Y, Inouye G, Tahara Y, Kondo K. On the chemical structure of menaquinones with the tetrahydrogenated isoprenoid side chain. Biochim Biophys Acta. 1977; 486:195–203.
4. Ohtsuka T, Itezono Y, Nakayama N, Kurano M, Nakada N, Tanaka H, et al. Structural elucidation of arthrobacilins A, B and C, structurally unique secondary metabolites of a microorganism. Tetrahedron Lett. 1992; 33:2705–2708.
5. Gomez JS, Shaikhet M, Loganathan AK, Darnowski MG, Boddy CN, McMullin DR, et al. Characterization of arthropeptide B, an antifungal cyclic tetrapeptide from *Arthrobacter humicola*. J Chem Ecol. 2023; 49:528–536.
6. Yu X, Jiang K, Zhang W, Dong S, Wu Y, Zhang G, et al. Purification, identification, and properties of a novel carotenoid produced by *Arthrobacter* sp. QL17 isolated from Mount Qomolangma. Antioxidants (Basel). 2022; 11:1493.
7. Ramlawi S, Abusharkh S, Carroll A, McMullin DR, Avis TJ. Biological and chemical characterization of antimicrobial activity in *Arthrobacter* spp. isolated from disease-suppressive compost. J Basic Microbiol. 2021; 61:745–756.
8. Gurusinghe S, Brooks TL, Barrow RA, Zhu X, Thotagamuwa LA, Dennis PG, et al. Technologies for the selection, culture and metabolic profiling of unique rhizosphere microorganisms for natural product discovery. Molecules. 2019; 24:1955.
9. Fu P, Macmillan JB. Thiasporines A-C, thiazine and thiazole derivatives from a marine-derived *Actinomycetospora chlora*. J Nat Prod. 2015; 78:548–551.
10. Saito S, Oku N, Igarashi Y. Mycetoindole, an N-acyl dehydrotryptophan with plant growth inhibitory activity from an actinomycete of the genus *Actinomycetospora*. J Antibiot. 2022; 75:44–47.
11. Jia J, Wang X, Sang J, Li Z, Lin S, Deng Z, et al. An N-N linked dimeric indole alkaloid from the marine sponge-associated rare actinomycetes *Kocuria* sp. S42. Nat Prod Res. 2023; 37:3647–3653.
12. Zhao G, Kosek D, Liu HB, Ohlemacher SI, Blackburne B, Nikolskaya A, et al. Structural basis for a dual function ATP grasp ligase that installs single and bicyclic ω-Ester macrocycles in a new multicore RiPP natural product. J Am Chem Soc. 2021; 143:8056–8068.
13. Brinkmann S, Kurz M, Patras MA, Hartwig C, Marner M, Leis B, et al. Genomic and chemical decryption of the Bacteroidetes phylum for its potential to biosynthesize natural products. Microbiol Spectr. 2022; 10:e02479-21.
14. Somanadhan B, Kotturi SR, Yan Leong C, Glover RP, Huang Y, Flotow H, et al. Isolation and synthesis of falcitidin, a novel myxobacterial-derived acyltetrapeptide with activity against the malaria target falcipain-2. J Antibiot. 2013; 66:259–264.
15. Haedar JR, Yoshimura A, Wakimoto T. New variochelins from soil-isolated *Variovorax* sp. H002. Beilstein J Org Chem. 2024; 20:692–700.

16. Ivanova AA, Kulichevskaya IS, Merkel AY, Toshchakov S V., Dedysh SN. High diversity of Planctomycetes in soils of two lichen-dominated sub-arctic ecosystems of Northwestern Siberia. Front Microbiol. 2016; 7:2065.
17. Assih EA, Ouattara AS, Thierry S, Cayol JL, Labat M, Monroy O. *Bosea minatitlanensis* sp. nov., a strictly aerobic bacterium isolated from an anaerobic digester. Int J Syst Evol Microbiol. 2003; 53:1247–1251.
18. Kanso S, Patel BKC. Microvirga subterranea gen. nov., sp. nov., a moderate thermophile from a deep subsurface Australian thermal aquifer. Int J Syst Evol Microbiol. 2003; 53:401–406.
19. De Meyer SE, Willems A. Multilocus sequence analysis of *Bosea* species and description of *Bosea lupini* sp. nov., Bosea lathyri sp. nov. and *Bosea robiniae* sp. nov., isolated from legumes. Int J Syst Evol Microbiol. 2012; 62:2505–2510.
20. Severiano A, Pinto FR, Ramirez M, Carriço JA. Adjusted Wallace Coefficient as a Measure of Congruence between Typing Methods. J Clin Microbiol. 2011; 49:3997–4000.
